# A new geometric and mechanical verification device for medical LINACs

**DOI:** 10.1120/jacmp.v3i2.2581

**Published:** 2002-03-01

**Authors:** Keith T. Welsh, Robert A. Wlodarczyk, L. E. Reinstein

**Affiliations:** ^1^ Radiation Oncology Department State University of New York at Stony Brook 7028 SUNY Stony Brook New York 11794‐7028

**Keywords:** Linac QA, Mini‐Gard, Mechanical Verification

## Abstract

The goal of quality assurance (QA) for a radiation oncology medical LINAC is to maintain an acceptable level of equipment performance and reliability. The increasing complexity of Radiation Oncology equipment and treatment techniques have led to increased demands on the work load of the medical physicist. Regular testing needs to be as efficient as possible. Generally, the QA tests, as recommended by the AAPM Task Group 40 for medical LINACs, can be grouped into two categories: dosimetry and mechanical checks. A new QA device has been developed that facilitates many of the daily and monthly mechanical QA checks. Its efficiency and speed is achieved through a set of QA tools that are mounted on a single platform, which is designed to fit into the accessory mount of the medical LINAC. Named Mini‐GARD (MG), it verifies the accuracy of the digital readouts for gantry angles, collimator angles, and field sizes. It also tests crosshair position, the optical distance indicator (ODI), and patient setup laser alignment. It uses two calibrated digital levels for the gantry and collimator angle verification, an electronic tape measure for ODI verification, and a calibrated transparent projection scale for the remaining tests. This paper evaluates the stability and accuracy of the device in clinical tests over a period of a year. Results show that the MG is reliable and capable of measuring gantry and collimator angle constancy to ±0.3°, ODI constancy to ±0.05 cm, and field size accuracies to ±0.05 cm.

PACS number(s): 87.56.Fc

## INTRODUCTION

A reliable quality assurance (QA) program for a medical LINAC is necessary to ensure that the prescribed radiation dose is accurately and reproducibly delivered to the patient's target volume. It is of fundamental importance to test and verify the geometric and mechanical accuracy of every medical LINAC on a regular basis.^1^ ^–^ ^4^ The increasing complexity of both medical LINACs and patient treatment techniques has raised the QA measurement burden of the medical physicist, and thus the manpower needs of the institution. In order to help reduce this burden and increase efficiency, a new device, designated Mini‐GARD (Geometric Accuracy Radiotherapy Device) has been developed that facilitates many of the daily and monthly geometrical and mechanical parameter verifications as recommended by the AAPM Task Group 40.^1^ The Mini‐GARD (MG) is a lightweight assembly that slides into the accessory mount of the medical linear accelerator and can be used to test the following LINAC geometrical and mechanical systems: gantry and collimator angle readout, field sizes readout, optical distance indicator (ODI), crosshair centricity, and transverse and overhead patient setup lasers.

The MG achieves increased efficiency through a setup of a single fixed platform from which all measurements are made, thus eliminating the extra time and uncertainty associated with the use of multiple independent measurement devices. The purpose of this paper is to describe the designed uses of the MG, and to determine its accuracy and reproducibility in the clinical environment over the period of a year.

## DESCRIPTION

**Figure 1:**
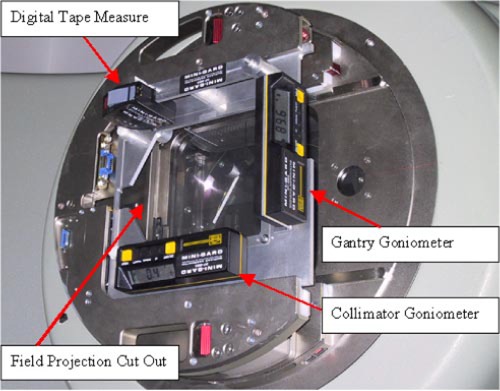
(Color) Picture of Mini‐Gard with components labeled.

**Figure 2:**
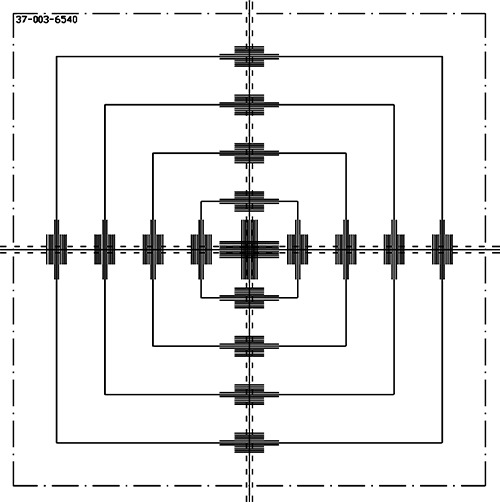
This is a diagram of the scribe lines on the acetate positioned over the cutout of the Mini‐Gard. Labeled are sizes of the boxes that project to field sizes of 5×5 through 25×25 at 100 SSD for an accessory tray position of 65.4 cm from the source.

The MG is composed of a flat (approximately $\frac{2}{3}-\text{cm}$ thick) aluminum support plate that fits into the accessory mount of the medical LINAC and has a $17\times 17{\text{cm}}^{2}$ aperture in the center. Mounted on this plate are two calibratable electronic digital goniometers for measurement of the gantry and collimator angles, a tape measure with an electronic digital readout for ODI verification, and a precision projection scale for light field size and laser alignment verifications (see Fig. 1.). Two electronic digital goniometers are mounted along adjacent edges of the plate and, once calibrated, have a precision of ±0.05° for a full 360° of rotation. When setup correctly they are oriented such that the sensitive rotational axis of one of the goniometers is parallel to the gantry rotational axis for measurement of the gantry angle, and the other goniometer has its axis parallel to the collimators rotational axis for measurement of the collimator angle. They are labeled in Fig. 1 as “Gantry Goniometer” and “Collimator Goniometer,” respectively. The electronic digital tape measure is mounted on an arm that swings out to position the tape measure at the center of the field. The digital tape measure has a precision of ±0.05 cm. When not in use, the tape measure arm locks outside of the field. The projection scale is marked on a sheet of acetate, which is mounted onto a sturdy adjustable transparent plastic sheet that spans the center aperture of the MG plate. A schematic of the projection scale on the acetate is shown in Fig. 2. The projection consists of a crosshair, to define the central axis, and outlines that represent the field sizes 5×5,10×10,15×15, and $20\times 20{\text{cm}}^{2}$ at 100‐cm SSD. In addition, there are tick marks in 1‐mm increment, for a projection measured at 100‐cm SSD, positioned along the cross hair lines to aid in the determination of field side errors. The position of this projection is easily maneuvered for initial alignment with LINAC's crosshair.

## METHOD AND RESULTS

### Gantry and collimator readout verification

The electronic digital goniometers must be calibrated against a reference standard before use. They are capable of storing a calibration fit to their entire 360° range from calibration points at four orientations representing the rotational angles of 0°, 90°, 180°, and 270°. We used a bubble level with an accuracy of ±0.1° as our reference standard. Calibration was performed with the MG in the accessory tray mount using the reference level at the angles of 0°, 90°, 180°, and 270° for gantry angle and at 0°, 90°, and 180° for the collimator angle. The individual calibrations at the above orientations within the accessory mount are required because the plane of the MG does not coincide with the gantry or collimator measurement plane. The discrepancy is a result of the sag between the accessory tray mount and the gantry and will change with gantry and collimator angle. Calibration of the MG with the gantry and collimator planes at each of the measurement angles takes into account all the individual discrepancies at those angles.

We tested the reliability and consistency of the MG digital goniometers, as well as the long‐term stability of their calibration by repeated comparisons to the reference standard for each of the seven gantry and collimator calibration positions. Note that because of the geometric orientation of the collimator the collimator angle must be measured with the gantry in the horizontal position. These tests were done on ten separate days spread throughout a year, without recalibration of the goniometers. On one occasion, these tests were repeated five times during a single day.

Table I shows a summary of the gantry and collimator test results. The table shows the average value at each of the reference positions, the average absolute deviation from the reference value as well as the maximum deviation from the reference value. The average absolute deviation is the average of the absolute values of the difference between the goniometer reading and the reference values and is a better representation of the device error than the standard deviation. The average readings at each orientation are within 0.15° of the reference value with the average absolute deviation equal to ∼0.1°. The maximum deviation from the reference values was 0.4°, but happened in only three of the 105 total measurements. Table II shows the results of five measurements repeated on the same day. It is evident from the table that the reproducibility of the digital goniometers within the same day is ≤0.1°.

**Table I:** The average measured value and average errors in the goniometer readings are shown for the gantry and collimator positions measured. Data is from ten different days over a period of a year. This table is constructed using the 105 data values. Aver. Abs. Dev. refers to the average discrepancy between the measured value and the reference level value regardless of sign.

Reference level position						
Goniometer	0°	90°	180°	270°	Average	Max Dev.
Gantry						
Average reading	–0.10	89.90	179.90	269.92		
Aver. Abs. Dev.	0.16	0.21	0.08	0.11	0.10	0.40
Collimator						
Average reading		89.86	180.13	269.98		
Aver. Abs. Dev.		0.23	0.16	0.13	0.17	0.40
Aver. Abs. Dev. (All)					0.12	

**Table II:** Shows the results of five tests performed on the same day.

		Gantry position				Collimator position	
Goniometer trial	90°	180°	270°	360°	90°	180°	270°
#1	90.1	179.9	270.0	359.9	89.7	179.9	269.9
#2	89.9	180.0	269.9	360.0	89.7	179.7	269.7
#3	89.9	180.0	269.9	359.9	89.7	179.8	269.7
#4	89.9	180.0	269.9	359.9	89.6	179.7	269.8
#5	89.9	180.0	269.9	360.0	89.7	179.7	269.8
Average	89.9	180.0	269.9	359.9	89.7	179.8	269.8
Aver. Abs. Dev.	0.10	0.02	0.08	0.06	0.32	0.24	0.22

### ODI verification using the electronic tape measure

**Figure 3:**
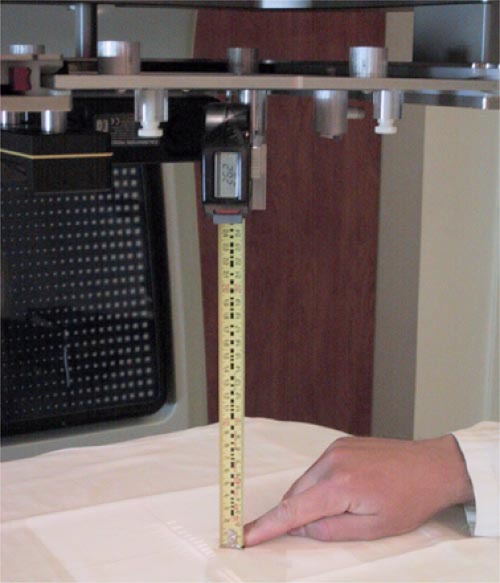
(Color) Mini‐Gard showing the operation of the electronic tape measure.

The electronic digital tape measure is composed of a standard tape measure with an optical reader that interprets encoded markings on the tape to determine and display the distance to a precision of ±0.05 cm. It indicates the distance from a reference point on the MG to the reference

surface (MSD). The tape measure is mounted on an arm that is locked outside the aperture in the base plate when not in use and swings into the central axis of the beam line for measurement of the MSD as shown in Figs. 1 and 3. The MSD is related to the SSD by an offset distance equal to the distance from the radiation source to the MG reference point and is designated the source to MG distance (SMD). The SMD is initially established for each LINAC and MG by the user with the help of SSD calibration rods, which are routinely supplied by the accelerator manufacturer. Subsequential verification of ODI distance is performed by measuring the MSD and applying the relation SSD=MSD+SMD. We tested the reproducibility of MG's SSD measurements by verifying the MSD setup using the distance calibration rods on ten separate days spanning a period of a year, as well as by multiple measurements recorded on the same day of an identical setup. Using the SMD calibrated at an SSD of 100 cm, we also calculated the SSD values as measured with the MG for the four different SSDs of 80, 90, 100, and 110 cm and compared them to the ODI rods.

The average measured MSD from 40 measurements taken on ten different days spanning a period of a year for an SSD of 100 cm was 25.74 cm with a standard deviation of only 0.05 cm. All measurements made had a MSD reading that varied between 25.8 cm and 25.7 cm. The results of the SSD's measured with the MG are shown in Table III. For each SSD, ten MSD measurements were recorded.

**Table III:** SSD as determined by the MG digital table measure.

Calibrated SSD (cm)	MG reading (cm)
80	80.0
90	90.1
100	100.0

### Crosshair, field size, and laser alignment verification

For the MG initialization of the precision projection scale, a conventional, independent method must be used to assure that the projected crosshair shadow on the LINAC is properly centered. The MG is then configured by adjusting the position of the acetates so that the crosshair on the acetate coincides with the projection of the LINAC's crosshair. The ability to use the MG to verify the LINAC's crosshair is dependent on the reproducibility of the MG within the accessory tray and the sag of the accessory mount on the LINAC. We tested the variation of the MG's crosshair projection position within our LINACs by recording the projected position of the MG's crosshair at an SSD of 100 cm for numerous insertions of the MG into the accessory tray. All distances were measured with a calibrated ruler that had 0.05‐cm scale. On a single day the reproducibility of the cross hair center was determined to be better than ±0.025 cm.

The medical LINAC's light field sizes are measured and its digital readouts verified by comparing its collimated light field with the calibrated projection scale from the MG acetate. The MG is designed such that its scale projects field sizes of 5×5,10×10,15×15, and $20\times 20{\text{cm}}^{2}$ and tick marks spaced 0.1 cm apart along the crosshairs at a SSD of 100 cm. The actual collimator light field size can then be easily measured by observing the shadow of the collimators against the scale. We verified the size of the field outlines on the acetate, as well as their projected sizes at 100‐cm SSD. The acetate scale dimensions were measured in order to verify the accuracy of the field size templates; the projections at 100 cm SSD were measured in order to assess their readability.

The field sizes measured on the acetate are shown in Table IV. The values have been multiplied by 100/64.5 in the table to give the values projected to a surface at a SSD of 100 cm. The uncertainties of measurements made on the acetate were estimated to be ±0.025 cm. The errors in the field sizes on the acetate projected to a SSD of 100 cm were ±0.04 cm. Measurements made of the actual projection at a SSD of 100 cm showed similar errors of ±0.05 cm, which is slightly higher because of the artifacts from the light bulb size and light transmission through the transparent material of the MG.

**Table IV:** The field sizes as measured on the acetate. Values are multiplied by 100/64.5 to give values projected at a SSD of 100 cm. Estimated uncertainties are ±0.04 cm.

	Measured field sizes on acetate projected to a SSD of 100 cm		
Field size (cm)	Width (cm)		Height (cm)
5×5	4.98	×	4.98
10×10	9.98	×	9.99
15×15	14.97	×	14.96
20×20	19.98	×	19.96

The laser alignment tests are performed with the gantry precisely rotated to the inverted vertical position for the overhead laser and the two horizontal positions for the right and left‐side transverse lasers. The laser alignment constancy is evaluated by observing the position of the laser lines on the coordinate system marked on the acetate of the MG. The initial baseline position of the laser on the acetate is established with the laser aligned using conventional methodologies, and with the gantry set to 0°, 90°, and 270° using the MG goniometers. The verification of the laser position is tested by measuring the laser's displacement from these initial calibrated positions.

Because the position of the lasers' projected cross on the MG acetate are dependent on the angular position of the gantry, any deviation of the gantry angle will cause errors in the position of the lasers' projections on the MG acetate. This error is given by

(1) Δd=Asin(Δθ),

where Δ*d* is the apparent displacement of the laser from the initial calibration position, Δθ is the error in the gantry position, and *A* is the distance from the isocenter to the accessory tray. For our LINAC A=34.6 cm. From the gantry goniometer results in Table I, the average deviation of gantry angular position is 0.12°. Then, from Eq. (1), the average error in the position of laser projected cross on the acetate will be 0.8 mm. The consequence of this is discussed below.

## DISCUSSION

The above experiments show that once calibrated a single MG device is well suited for daily and monthly QA tests on an individual LINAC. The average deviation of the gantry and collimator tests with the MG was 0.12° from the reference angle and is within the TG‐40 suggested tolerance of ±1°. However, over the course of a year the error was not randomly scattered about an average, but drifted over time. This drift reached a maximum error of 0.3° after three months. Because of this drift, we suggest that the goniometers be recalibrated after three‐six months.

The 1‐mm precision of the MG's electronic tape measure is within the limits required for the TG‐40 suggested ODI verification tolerance (±0.2 cm). Its SSD readings were stable and reproducible to within ±0.05 cm over a 12 month period with an accuracy of ±0.06 cm.

The reproducibility of the MG's positioning of the field size and crosshair are also within the ±2 mm tolerance suggested by TG‐40. Its reproducibility, however, is dependent on the slack within the LINAC's accessory tray, so the variance of the MG's crosshairs should be determined for an individual machine.

The small uncertainty of the gantry angle, determined by the MG, may lead to inadequate precision in the verification of the patient positioning lasers. The average and maximum gantry goniometer angle errors of 0.12° and 0.4° results in errors in the angular position of the laser on the acetate of 0.08 cm and 0.24 cm, respectively. Thus, on average the uncertainty caused by the gantry angle error would be acceptable for the TG‐40 suggested tolerance of ±0.2 cm, but the larger errors in the gantry angle could produce incorrect results.

## CONCLUSION

The Mini‐GARD is an alternative QA device for measuring the geometric parameters associated with standard daily and monthly QA protocols. We have quantitatively tested its accuracy and determined that it is well suited for most of its purposed uses. The uncertainties in the gantry angle, collimator angle, and ODI verification are well within the tolerances needed for daily and monthly protocols. The crosshairs and field size verification are also within acceptable tolerances for most LINACs, but should be verified before use. The usefulness of the laser alignment test depends on the tolerances established at each institution and should be carefully evaluated.
